# Trends of kidney cancer burden from 1990 to 2019 in European Union 15 + countries and World Health Organization regions

**DOI:** 10.1038/s41598-022-25485-8

**Published:** 2022-12-26

**Authors:** Chinmay Jani, Nour Abdallah, Christian Mouchati, Ruchi Jani, Rajesh Sharma, Padmanabh Bhatt, Georgina Hanbury, Justin Salciccioli, Harpreet Singh, Joseph Shalhoub, Rana R. McKay, Dominic C. Marshall

**Affiliations:** 1 Department of Internal Medicine, Mount Auburn Hospital, 330 Mount Auburn St, Cambridge, MA 02138 USA; 2 Harvard Medical School, Boston, MA USA; 3 Department of Urology Research, Glickman Urological and Kidney Institute, Cleveland Clinic, Cleveland, OH USA; 4 Department of Pediatrics, Case Western Reserve University, Cleveland, OH USA; 5 University Hospitals Cleveland Medical Center, Cleveland, OH USA; 6 Smt. NHL Municipal Medical College, Ahmedabad, Gujarat India; 7 NIT Kurukshetra, Kurukshetra, India; 8 Imperial College HealthCare NHS Trust, London, UK; 9 Division of Pulmonary and Critical Care, Brigham and Women’s Hospital, Boston, MA USA; 10 Department of Medicine, Division of Pulmonary and Critical Care, Medical College of Wisconsin, Milwaukee, WI USA; 11 Academic Section of Vascular Surgery, Department of Surgery and Cancer, Imperial College London, London, UK; 12 University of California San Diego, San Diego, CA USA; 13 Department of Surgery and Cancer, Imperial College London, London, UK

**Keywords:** Cancer, Medical research, Oncology

## Abstract

In recent decades, variability in the incidence and mortality of kidney cancer (KC) has been reported. This study aimed to compare trends in incidence, mortality, and disability-adjusted life years (DALY) of KC between the European Union (EU) 15 + countries and 6 World Health Organization (WHO) regions. The data of KC Age-standardized incidence rates (ASIRs), age-standardized mortality rates (ASMRs), and age-standardized DALYs were extracted from the Global Burden of Disease database. Joinpoint regression was employed to examine trends. From 1990 to 2019, the ASIR increased in most countries except for Luxembourg (males), the USA (females) and Austria and Sweden (both sexes). ASIR increased across all 6 WHO regions for both sexes except for females in Americas. The ASMR increased in 10/19 countries for males and 9/19 for females as well across most WHO regions. The mortality-to-incidence ratio (MIR) decreased in all countries and WHO regions. Trends in DALYs were variable across countries and WHO regions. While the incidence and mortality from KC rose in most EU15 + countries and WHO regions from 1990 to 2019, the universal drop in MIR suggests an overall improvement in KC outcomes. This is likely multifactorial, including earlier detection of KC and improved treatments.

## Introduction

Kidney Cancer (KC) is ranked among the most common cancers diagnosed worldwide and is increasing in incidence^1^. In 2020, around 400,000 new diagnoses of KC were estimated worldwide, with 180,000 estimated deaths^2^. The main histologic type of KC is renal cell carcinoma (RCC), accounting for 90% of the incident cases and most of the KC morbidity and deaths^3^. Subtypes of RCC include clear cell, papillary, and chromophobe histology in 70%, 15%, and 5% of the cases, respectively^4^. The strongest risk factors related to KC are age, gender, obesity, smoking, hypertension, and chronic kidney diseases^4–9^.

In recent decades, significant variability in incidence and mortality of KC was noted worldwide^5^, with an average age-standardized incidence rate (ASIR) of 4.6 per 100,000 noted in 2020 and an average age-standardized mortality rate (ASMR) of 1.8 per 100,000^10^. The incidence was reported twice higher in males than females and is more common in developed countries than in developing countries^10,11^. For instance, Northern America had the highest ASIR of 12.2 per 100,000, followed by Northern Europe, Australia, and New Zealand (10.3), and Central and Eastern Europe (3.4 per 100,000) had the highest ASMR, followed by Western Europe (2.8), Northern Europe (2.7), and Australia and New Zealand (2.4)^10^.

While the incidence of KC has been increasing in the past decades, mortality is decreasing in Western Europe and most Northern European countries and the United States of America (USA) and Australia^11^. One possible reason behind the stability of mortality rates would be earlier detection, improved surgical and ablative techniques, and improved systemic treatment options for patients with locally advanced or metastatic RCC^12^. Furthermore, the ever-increasing use of imaging, including computed tomography (CT) scan, has led to the identification of incidental asymptomatic renal masses. This has resulted in stage migration with detection of earlier stage disease and reductions in mortality rates in American and European regions^13,14^.

This study aims to compare the trends in KC among countries of the European Union (EU) 15 + cohort and between 6 WHO regions during the period ranging from 1990 to 2019. The following 19 countries are unitedly included in the EU15 + grouping: Australia, Austria, Belgium, Canada, Denmark, Finland, France, Germany, Greece, Ireland, Italy, Luxembourg, Netherlands, Norway, Portugal, Spain, Sweden, the United Kingdom (UK), and the USA. The WHO regions include the African Region (AFR), Region of the Americas (AMR), South-East Asian Region (SEAR), European region (EUR), Eastern Mediterranean Region (EMR), and Western Pacific Region (WPR). The EU15 + has been previously utilized in comparative analyses of trends in other diseases^15,16^, as it forms a comparable group of countries. Data were extracted from the Global Burden of Disease (GBD) Study to evaluate the trends in KC mortality, incidence, and disability-adjusted life years (DALYs) globally, in WHO regions, and in the EU15 + countries from 1990 to 2019. Joinpoint regression analysis was used to describe incidence, mortality, and DALYs changes and the analysis of significant trends present in the studied period.

## Methods

### Characteristics of the data source

This observational analysis of KC among EU15 + countries was performed using data from the GBD database. Previous publications describe the exact GBD methodology in greater detail^17,18^, and we have used this database to describe trends in peripheral arterial disease^16^, abdominal aortic aneurysm^19^, and lower extremity amputation^20^. Data sets used by the GBD collaborators include insurance, admission, and outpatient encounter data, and published systematic reviews, among others. For KC, the GBD maps all mortality and incidence data related to the International Classification of Diseases (ICD) codes (codes C64-C65 and D41.0, D41.1 from ICD-10, and codes 189.1, 189.1 from ICD9). These data are then combined by Bayesian meta-regression with the DisMod-MR 2.19 tool^21^ that analyses, adjust for bias, and produces disease estimates with uncertainty intervals. GBD has different mappings of ICD codes based on incidence and mortality.

Mortality data is collected from vital registration sources, verbal autopsy reports, and surveillance data and entered the GBD cause-of-death database. The quality of mortality data from each country is rated by the GBD methodology in a 5-star system by location-year to assist in the reader’s comprehension of the reliability of the cause of death data. The EU15 + countries have been previously analyzed this way, with 10 of 19 countries scoring five stars (85–100% completeness of mortality data), and the remaining nine countries scored four stars (65–84% completeness of mortality data).

### Handling of the GBD data

We extracted age-standardized incidence rates (ASIRs), age-standardized mortality rates (ASMRs), and age-standardized disability-adjusted life years (DALYs) for KC from EU15 + countries between 1990 and 2019 using the dedicated GBD Study results tool (http://ghdx.healthdata.org/gbd-results-tool). Age-standardized rates are used to account for the age structures of each country and are expressed per 100,000 person-years. The method used by the GBD involves calculating a standard population by GBD.

We calculated absolute and relative changes in ASIRs, ASMRs, and DALYs between 1990 and 2019 for each sex in each country and each region. The mortality-to-incidence ratios (MIRs) were calculated by dividing ASMR by ASIR for each year (1990 and 2019) in all EU15 + countries and 6 WHO regions. MIRs compare disease burden by normalizing mortality to incidence, and disability-adjusted life-years incorporate morbidity and mortality figures to calculate the number of years lived with and lost from a disability. The WHO also uses this metric to indicate the overall burden of disease on a health-system^11^. These measures facilitate our understanding of KC’s varying temporal impact. Global and different WHO region mean trends are also reported for comparison.

The datasets analysed during the current study are available in the GBD repository, http://ghdx.healthdata.org/gbd-results-tool.

### Statistical analysis

Joinpoint Command Line Version 4.5.0.1 was used to apply a Joinpoint regression analysis to the incidence, mortality, and DALYs data (provided for free by the United States National Cancer Institute Surveillance Research Program)^22^. The software observes trends in the data over the time studied and connects these trends with the simplest model possible on a logarithmic scale. It produces periods where significant trend points have been identified. It will identify specific inflection points in the overall trends and provide a robust estimate of each country's changing trends. The simplest model has no Joinpoints and represents a straight line. As more Joinpoints are added, each is tested for significance using a Monte Carlo permutation method. The Joinpoint software also computed an estimated annual percentage change (EAPC) (with 95% confidence intervals) for each Joinpoint line segment and tested for significance. The result of the analyses is a series of statistically significant Joinpoints for each country, with each trend (either positive or negative) represented by a potentially significant EAPC. This allows a thorough assessment of temporal trends and allows for inter-country comparability.

## Results

Trends in KC among EU15 + countries were analyzed from 1990 to 2019. Age-adjusted incidence and mortality rates, mortality-incidence ratios, and age-standardized DALYs were compared in this study.

### Trends in KC ASIR, 1990–2019

Across the study period, there was a rise in ASIR globally for both males (+ 38.4%) and females (+ 13.5%). There was a similar trend across all six regions, with the Western Pacific region seeing the greatest increase in males (+ 142.8%), while the Eastern Mediterranean region saw the greatest increase in females (+ 98.4%). The Americas had the smallest increase in ASIR for males (+ 16.4%) and was the only region to observe a fall in ASIR for females (− 6.9%).

Amongst EU 15 + countries, Denmark had the greatest increase in ASIR for both males and females, + 89.3 and 82.8%, respectively. Austria was among the only three countries witnessing a fall in ASIR for males and saw the greatest fall (− 18.0%). Sweden saw the greatest fall in ASIR for females (− 24.0%).

**Figure 1:**
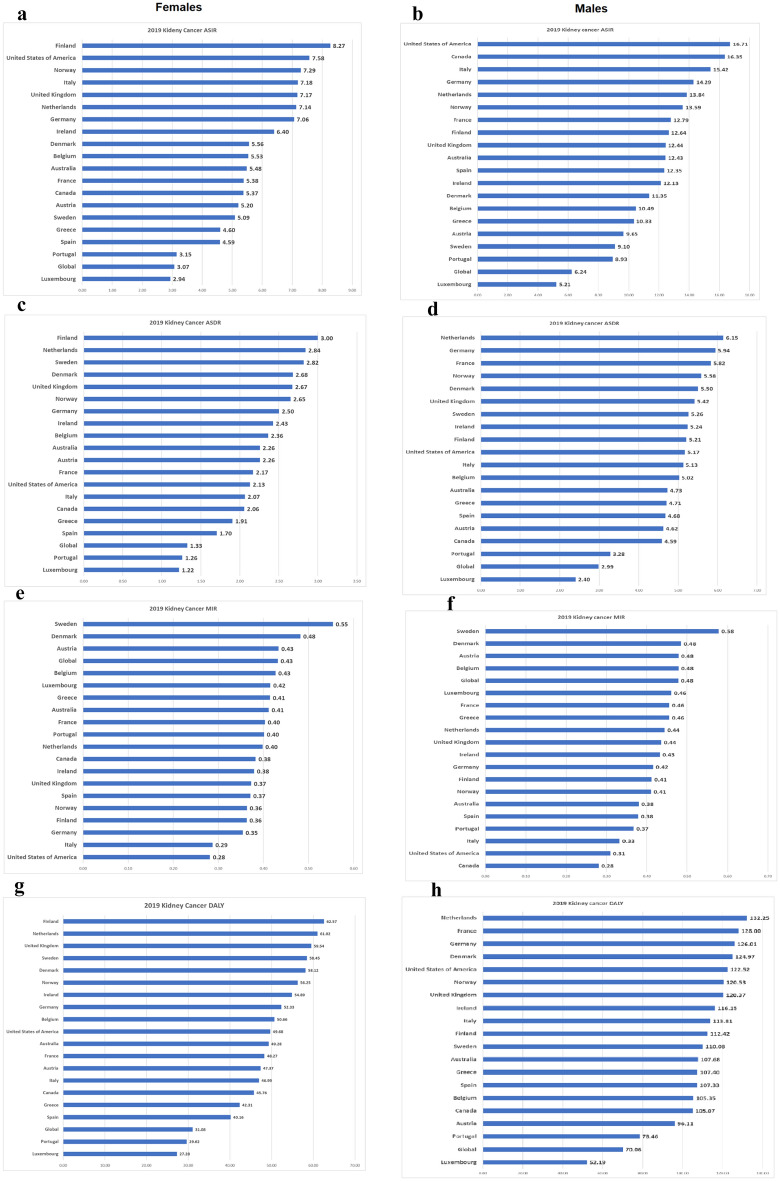
Age-standardized incidence rates (ASIR) for females (**a**) and males (**b**), age-standardized mortality rates for females (ASMR) (**c**) and males (**d**), mortality-to-incidence ratios (MIR) for females (**e**) and males (**f**), and disability-adjusted life years (DALYs) for females (**g**) and males (**h**) for kidney cancer (KC) for EU 15 + countries in 2019. All indices are per 100,000 population.

**Figure 2:**
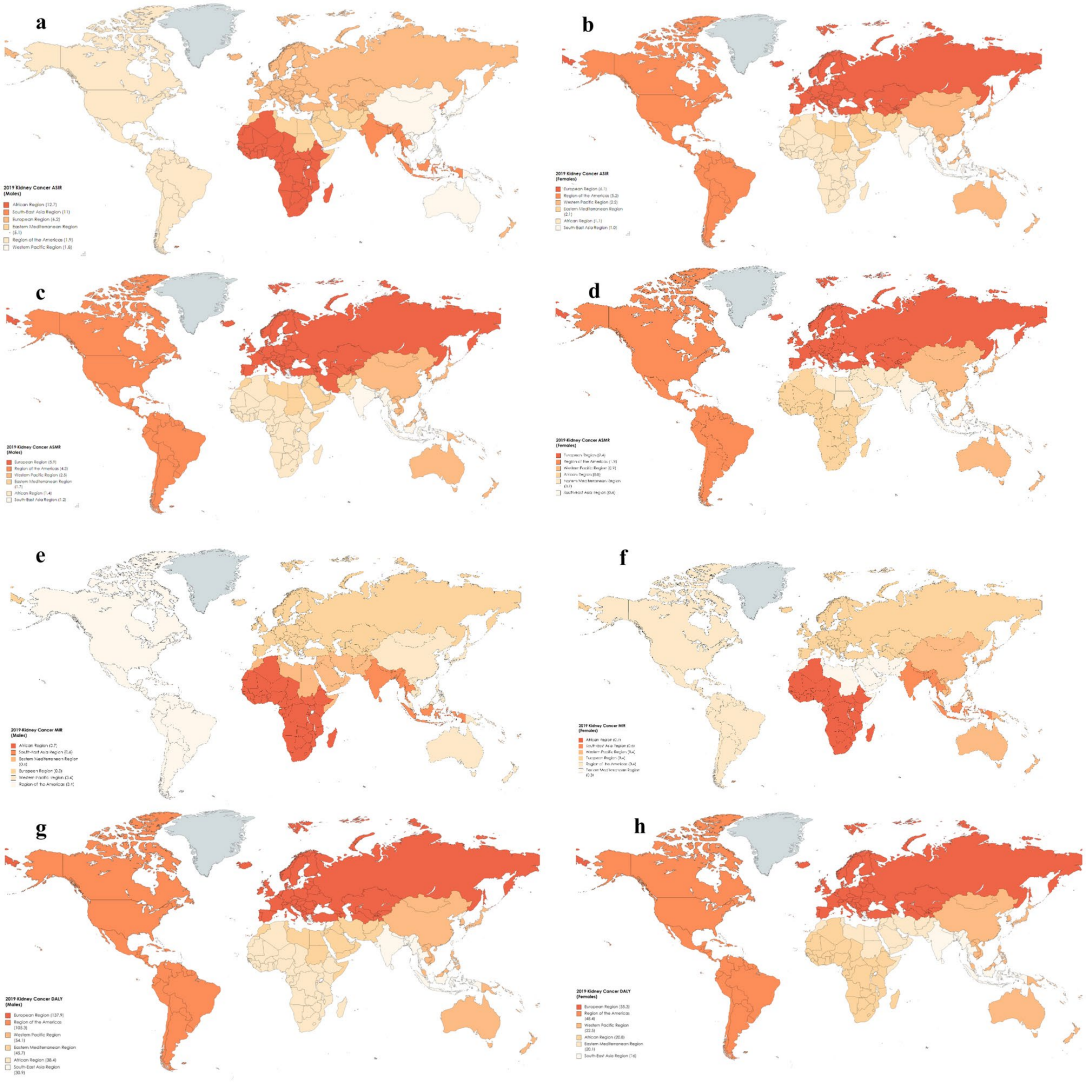
Age-standardized incidence rates (ASIR) for males (**a**) and females (**b**), age-standardized mortality rates for males (ASMR) (**c**) and females (**d**), mortality-to-incidence ratios (MIR) for males (**e**) and females (**f**) and disability-adjusted life years (DALYs) for males (**g**) and females (**h**) for kidney cancer (KC) for WHO regions in 2019. All indices are per 100,000 population. The figure is created by Dr. Chinmay Jani with mapchart.net (URL: https://www.mapchart.net/) and is licensed under the Creative Commons Attribution-ShareAlike 4.0 International License.

Tables 1 and 2 and, Figs. 1, 2 depict gender-specific trends in KC ASIR across EU 15 + countries and 6 WHO regions.

**Table 1:** 1990 and 2019 female age-standardized mortality rates (ASMRs), age-standardized incidence rates (ASIRs), mortality-to-incidence ratios (MIR), and disability-adjusted life years (DALYs), with associated percentage changes, for KC in the European Union 15 + countries and WHO regions.

Country/Region	DALY			ASIR			ASDR			MIR		
Female	1990	2019	% change	1990	2019	% change	1990	2019	% change	1990	2019	% change
Global	34.30	31.08	− 9.39	2.70	3.07	13.54	1.36	1.33	− 2.29	0.50	0.43	− 13.94
Australia	64.82	49.28	− 23.97	5.11	5.48	7.35	2.80	2.26	− 19.23	0.55	0.41	− 24.76
Austria	80.60	47.37	− 41.23	6.57	5.20	− 20.80	3.52	2.26	− 35.84	0.54	0.43	− 18.98
Belgium	62.61	50.66	− 19.09	5.01	5.53	10.54	2.70	2.36	− 12.40	0.54	0.43	− 20.75
Denmark	44.64	58.12	30.20	3.04	5.56	82.80	1.94	2.68	37.75	0.64	0.48	− 24.65
Finland	64.31	62.57	− 2.71	6.17	8.27	33.99	2.86	3.00	4.90	0.46	0.36	− 21.71
France	56.41	48.27	− 14.43	4.41	5.38	22.06	2.36	2.17	− 8.20	0.54	0.40	− 24.79
Germany	66.65	52.33	− 21.49	6.61	7.06	6.88	2.66	2.50	− 5.87	0.40	0.35	− 11.93
Greece	42.92	42.31	− 1.41	3.60	4.60	27.81	1.88	1.91	1.50	0.52	0.41	− 20.58
Ireland	50.40	54.89	8.90	3.92	6.40	63.02	2.03	2.43	19.68	0.52	0.38	− 26.58
Italy	54.77	46.99	− 14.20	6.03	7.18	19.07	2.12	2.07	− 2.63	0.35	0.29	− 18.23
Luxembourg	36.46	27.28	− 25.19	2.86	2.94	2.79	1.53	1.22	− 20.42	0.54	0.42	− 22.58
Netherlands	64.32	61.02	− 5.13	5.42	7.14	31.78	2.70	2.84	5.16	0.50	0.40	− 20.20
Norway	59.62	56.25	− 5.65	5.41	7.29	34.62	2.50	2.65	6.08	0.46	0.36	− 21.20
Portugal	39.47	29.62	− 24.96	2.67	3.15	17.77	1.42	1.26	− 11.17	0.53	0.40	− 24.57
Spain	38.25	40.16	4.99	3.06	4.59	50.02	1.51	1.70	12.58	0.49	0.37	− 24.95
Sweden	95.03	58.45	− 38.50	6.70	5.09	− 24.00	4.05	2.82	− 30.43	0.61	0.55	− 8.46
United Kingdom	58.71	59.54	1.41	5.22	7.17	37.38	2.35	2.67	13.64	0.45	0.37	− 17.28
Canada	43.79	45.76	4.48	4.01	5.37	34.03	1.78	2.06	15.24	0.45	0.38	− 14.02
United States of America	61.86	49.68	− 19.69	7.69	7.58	− 1.46	2.40	2.13	− 11.19	0.31	0.28	− 9.87
African Region	16.61	20.84	25.47	0.77	1.15	48.61	0.59	0.78	32.47	0.76	0.68	− 10.87
Region of the Americas	63.27	48.39	− 23.52	5.58	5.19	− 6.88	2.33	1.95	− 16.40	0.42	0.37	− 10.23
South-East Asia Region	12.83	15.99	24.67	0.70	1.05	49.59	0.47	0.59	25.53	0.68	0.57	− 16.08
European Region	54.33	55.30	1.78	4.58	6.14	33.94	2.09	2.37	13.08	0.46	0.39	− 15.58
Eastern Mediterranean Region	14.74	20.09	36.29	1.06	2.10	98.37	0.51	0.73	43.05	0.48	0.35	− 27.89
Western Pacific Region	21.07	22.54	7.00	1.29	2.21	70.78	0.73	0.90	23.21	0.57	0.41	− 27.85

All indices are per 100,000 population.

**Table 2:** 1990 and 2019 male age-standardized mortality rates (ASMRs), age-standardized incidence rates (ASIRs), mortality-to-incidence ratios (MIR), and disability-adjusted life years (DALYs), with associated percentage changes, for KC in the European Union 15 + countries and WHO regions.

Country/Region	DALY			ASIR			ASDR			MIR		
Males	1990	2019	% change	1990	2019	% change	1990	2019	% change	1990	2019	% change
Global	61.96	70.06	13.08	4.51	6.24	38.37	2.50	2.99	19.35	0.55	0.48	− 13.74
Australia	113.70	107.68	− 5.29	9.76	12.43	27.40	4.86	4.73	− 2.71	0.50	0.38	− 23.63
Austria	158.91	96.11	− 39.52	11.77	9.65	− 18.00	6.99	4.62	− 33.85	0.59	0.48	− 19.32
Belgium	115.47	105.35	− 8.76	8.53	10.49	22.85	5.12	5.02	− 1.99	0.60	0.48	− 20.22
Denmark	90.17	124.97	38.59	6.00	11.35	89.34	3.89	5.50	41.65	0.65	0.48	− 25.19
Finland	143.44	112.42	− 21.63	11.63	12.64	8.69	6.01	5.21	− 13.27	0.52	0.41	− 20.21
France	140.90	128.00	− 9.16	10.12	12.79	26.41	6.03	5.82	− 3.49	0.60	0.46	− 23.65
Germany	137.13	126.01	− 8.11	11.45	14.29	24.75	5.61	5.94	5.96	0.49	0.42	− 15.06
Greece	94.83	107.40	13.26	7.21	10.33	43.41	4.10	4.71	14.84	0.57	0.46	− 19.93
Ireland	102.69	116.15	13.10	7.30	12.13	66.10	4.32	5.24	21.30	0.59	0.43	− 26.97
Italy	130.24	113.81	− 12.62	12.65	15.42	21.87	5.24	5.13	− 2.18	0.41	0.33	− 19.73
Luxembourg	74.53	52.19	− 29.97	5.38	5.21	-3.02	3.23	2.40	− 25.66	0.60	0.46	− 23.34
Netherlands	129.77	132.25	1.91	10.13	13.84	36.61	5.54	6.15	10.99	0.55	0.44	− 18.75
Norway	125.44	120.53	− 3.92	10.08	13.59	34.78	5.52	5.58	1.05	0.55	0.41	− 25.02
Portugal	78.58	78.46	− 0.16	5.71	8.93	56.42	2.95	3.28	11.10	0.52	0.37	− 28.97
Spain	88.73	107.33	20.96	7.85	12.35	57.33	3.58	4.68	30.73	0.46	0.38	− 16.91
Sweden	155.36	110.08	− 29.14	10.42	9.10	− 12.64	6.83	5.26	− 22.87	0.66	0.58	− 11.71
United Kingdom	118.17	120.37	1.86	8.79	12.44	41.48	4.90	5.42	10.62	0.56	0.44	− 21.81
Canada	89.91	105.07	16.86	11.30	16.35	44.63	3.69	4.59	24.47	0.33	0.28	− 13.94
United States of America	133.60	122.52	− 8.29	14.90	16.71	12.10	5.26	5.17	− 1.61	0.35	0.31	− 12.23
African Region	30.80	38.44	24.79	1.23	1.84	49.80	1.00	1.38	37.92	0.81	0.75	− 7.94
Region of the Americas	103.09	105.28	2.13	9.47	11.02	16.36	3.99	4.32	8.32	0.42	0.39	− 6.91
South-East Asia Region	19.93	30.88	54.94	0.98	1.88	90.97	0.73	1.20	62.84	0.75	0.64	− 14.73
European Region	124.62	137.89	10.65	8.88	12.65	42.53	4.93	5.86	19.04	0.55	0.46	− 16.48
Eastern Mediterranean Region	25.89	45.71	76.56	1.20	2.79	131.92	0.93	1.74	87.82	0.77	0.62	− 19.01
Western Pacific Region	33.10	54.08	63.36	2.11	5.12	142.81	1.29	2.28	76.55	0.61	0.44	− 27.29

All indices are per 100,000 population.

#### The Joinpoint analyses for ASIR in males and females are shown in Table 3 and Fig. 3

**Table 3:** Joinpoint analysis for KC age-standardized incidence rates in EU15 + countries and WHO regions for years 1990–2019 in males (A), females (B).

(A)												
Country/Region	Trend 1			Trend 2			Trend 3			Trend 4		
	Years	EAPC (95% CI)	“P-value”	Years	EAPC (95% CI)	“P-value”	Years	EAPC (95% CI)	“P-Value”	Years	EAPC (95% CI)	“P-value”
Global	1990–1994	2.6 (2.1–3.2)	< 0.001	1994–1997	0.8 (− 1–2.5)	0.371	1997–2009	1.6 (1.4–1.7)	< 0.001	2009–2019	0.1 (− 0.1–0.2)	0.203
Australia	1990–1999	2.4 (2.2–2.7)	< 0.001	1999–2003	− 1.7 (− 3.2–0.2)	0.025	2003–2007	3 (1.5–4.6)	0.001	2007–2019	− 0.3 (− 0.5–0.2)	0.001
Austria	1990–1999	− 1.4 (− 1.8–1.1)	< 0.001	1999–2019	− 0.5 (− 0.6–0.4)	< 0.001						
Belgium	1990–1996	− 0.2 (− 1.1–0.7)	0.645	1996–2002	4.3 (3–5.6)	< 0.001	2002–2019	− 0.4 (− 0.5–0.2)	0.001			
Denmark	1990–1996	9.1 (8–10.2)	< 0.001	1996–2009	1.5 (1.2–1.9)	< 0.001	2009–2019	− 0.5 (− 1–0)	0.034			
Finland	1990–1998	1.7 (1.4–2.1)	< 0.001	1998–2002	− 1.5 (− 3–0.1)	0.071	2002–2011	0.3 (0–0.7)	0.057	2011–2019	− 0.2 (− 0.6–0.1)	0.239
France	1990–2007	0.9 (0.8–1)	< 0.001	2007–2011	2.3 (0.7–4)	0.007	2011–2019	− 0.2 (− 0.5–0.2)	0.279			
Germany	1990–1995	4 (3.2–4.7)	< 0.001	1995–2019	0.1 (0–0.2)	0.002						
Greece	1990–1996	3.5 (2.8–4.3)	< 0.001	1996–2010	1.1 (0.9–1.3)	< 0.001	2010–2019	− 0.4 (− 0.8–0.1)	0.026			
Ireland	1990–1996	2.4 (1.5–3.3)	< 0.001	1996–2000	5.6 (3–8.3)	< 0.001	2000–2009	2.3 (1.7–2.9)	< 0.001	2009–2019	− 0.9 (− 1.3–0.5)	< 0.001
Italy	1990–2005	1.6 (1.5–1.8)	< 0.001	2005–2019	− 0.4 (− 0.6–0.3)	< 0.001						
Luxembourg	1990–2004	1 (0.8–1.1)	< 0.001	2004–2013	− 1 (− 1.4–0.7)	< 0.001	2013–2016	− 3.1 (− 5.9–0.1)	0.041	2016–2019	0.1 (− 1.4–1.6)	0.913
Netherlands	1990–2000	1.8 (1.4–2.3)	< 0.001	2000–2003	− 1.5 (− 7.3–4.7)	0.612	2003–2008	4.3 (2.3–6.3)	< 0.001	2008–2019	− 0.5 (− 0.9–0.1)	0.024
Norway	1990–2011	2.2 (2–2.3)	< 0.001	2011–2019	− 2.1 (− 2.6–1.5)	< 0.001						
Portugal	1990–1998	3.2 (2.5–3.9)	< 0.001	1998–2001	− 4.5 (− 10.3–1.7)	0.14	2001–2006	6 (3.9–8.1)	< 0.001	2006–2019	0 (− 0.3–0.3)	0.983
Spain	1990–1996	3.4 (3–3.8)	< 0.001	1996–2006	0.9 (0.6–1.1)	< 0.001	2006–2015	1.8 (1.6–2.1)	< 0.001	2015–2019	− 0.2 (− 1–0.5)	0.512
Sweden	1990–2007	− 1 (− 1.2–0.9)	< 0.001	2007–2019	0.1 (− 0.1–0.4)	0.348						
United Kingdom	1990–1995	2.6 (2–3.2)	< 0.001	1995–2006	1.3 (1.1–1.5)	< 0.001	2006–2019	0.6 (0.5–0.7)	< 0.001			
Canada	1990–2001	1.1 (0.9–1.3)	< 0.001	2001–2004	5.9 (2.7–9.2)	0.001	2004–2008	2.1 (0.5–3.7)	0.012	2008–2019	− 0.1 (− 0.4–0.1)	0.164
United States of America	1990–1994	2.6 (1.8–3.4)	< 0.001	1994–2002	0.5 (0.1–0.8)	0.006	2002–2006	− 0.9 (− 2–0.3)	0.138	2006–2019	0.1 (− 0.1–0.2)	0.394
African Region	1990–2013	1.5 (1.4–1.5)	< 0.001	2013–2019	1.2 (1–1.5)	< 0.001						
Region of the Americas	1990–1994	2 (1.4–2.6)	< 0.001	1994–2002	0.6 (0.3–0.8)	< 0.001	2002–2019	0.1 (0.1–0.2)	< 0.001			
South-East Asia Region	1990–1992	2 (− 0.9–5)	0.16	1992–1998	5 (4.3–5.7)	< 0.001	1998–2006	0.3 (− 0.1–0.7)	0.088	2006–2019	2.1 (1.9–2.2)	< 0.001
European Region	1990–1994	4.1 (3.3–4.9)	< 0.001	1994–1997	0.4 (− 2–2.9)	0.725	1997–2008	1.5 (1.3–1.7)	< 0.001	2008–2019	0.2 (0–0.3)	0.037
Eastern Mediterranean Region	1990–1996	2.3 (2–2.6)	< 0.001	1996–2001	3.8 (3.3–4.4)	< 0.001	2001–2011	2.9 (2.7–3)	< 0.001	2011–2019	3.1 (2.9–3.3)	< 0.001
Western Pacific Region	1990–1998	2.4 (2–2.8)	< 0.001	1998–2005	6.3 (5.7–6.9)	< 0.001	2005–2010	3.9 (2.8–5)	< 0.001	2010–2019	0.6 (0.3–1)	< 0.001

(B)												
Country/Region	Trend 1			Trend 2			Trend 3			Trend 4		
	Years	EAPC (95% CI)	“P-value”	Years	EAPC (95% CI)	“P-value”	Years	EAPC (95% CI)	“P-value”	Years	EAPC (95% CI)	“P-value”
Global	1990–1993	1.5 (0.8–2.3)	< 0.001	1993–2009	0.7 (0.6–0.7)	< 0.001	2009–2014	− 0.7 (− 1.1–0.2)	0.008	2014–2019	0 (− 0.3–0.4)	0.819
Australia	1990–1994	3.8 (2.5–5)	< 0.001	1994–2004	0.8 (0.4–1.1)	< 0.001	2004–2017	− 1.4 (− 1.6–1.2)	< 0.001	2017–2019	1.1 (− 2.6–4.9)	0.562
Austria	1990–2019	− 0.9 (− 1–0.9)	< 0.001									
Belgium	1990–1996	− 1.3 (− 2.2–0.3)	0.013	1996–2001	4.5 (2.6–6.4)	< 0.001	2001–2019	− 0.5 (− 0.7–0.3)	< 0.001			
Denmark	1990–1992	6.5 (2.8–10.3)	0.001	1992–1995	14.2 (10.2–18.3)	< 0.001	1995–1999	4.7 (2.8–6.5)	< 0.001	1999–2019	− 0.5 (− 0.6–0.4)	< 0.001
Finland	1990–2003	2.1 (1.9–2.4)	< 0.001	2003–2019	0 (− 0.2–0.2)	0.87						
France	1990–1999	1.2 (1–1.5)	< 0.001	1999–2007	− 0.4 (− 0.8–0.1)	0.026	2007–2014	1.5 (1–1.9)	< 0.001	2014–2019	0.2 (− 0.5–0.8)	0.586
Germany	1990–1995	2.3 (1.7–3)	< 0.001	1995–2019	− 0.3 (− 0.3–0.2)	< 0.001						
Greece	1990–1998	3.1 (2.7–3.6)	< 0.001	1998–2007	− 0.1 (− 0.5–0.3)	0.674	2007–2011	− 1.6 (− 3.4–0.3)	0.095	2011–2019	0.6 (0.2–1.1)	0.005
Ireland	1990–1995	1.6 (0.5–2.6)	0.006	1995–2006	3.8 (3.5–4.2)	< 0.001	2006–2019	0.1 (− 0.2–0.3)	0.501			
Italy	1990–2003	1.4 (1.2–1.5)	< 0.001	2003–2019	− 0.2 (− 0.3–0.1)	0.001						
Luxembourg	1990–2000	1.3 (1.1–1.5)	< 0.001	2000–2013	0.1 (− 0.1–0.2)	0.465	2013–2016	− 3.4 (− 6.1–0.6)	0.019	2016–2019	− 0.5 (− 1.9–0.9)	0.459
Netherlands	1990–2007	1.5 (1.2–1.8)	< 0.001	2007–2019	− 0.4 (− 0.9–0.1)	0.153						
Norway	1990–2002	2.8 (2.6–3)	< 0.001	2002–2011	1.2 (0.9–1.6)	< 0.001	2011–2017	− 2.5 (− 3.2–1.7)	< 0.001	2017–2019	0.4 (− 3–3.9)	0.809
Portugal	1990–1998	1.4 (1–1.8)	< 0.001	1998–2001	− 5.3 (− 8.9–1.6)	0.008	2001–2004	4.8 (0.9–8.9)	0.019	2004–2019	0.5 (0.3–0.6)	< 0.001
Spain	1990–1995	2.5 (1.9–3.1)	< 0.001	1995–2008	1.9 (1.7–2)	< 0.001	2008–2014	− 0.2 (− 0.7–0.4)	0.557	2014–2019	0.9 (0.4–1.5)	0.002
Sweden	1990–1999	− 0.6 (− 0.9–0.3)	0.001	1999–2002	− 3.1 (− 6.5–0.5)	0.084	2002–2019	− 0.8 (− 0.9–0.7)	< 0.001			
United Kingdom	1990–1995	2.2 (1.9–2.6)	< 0.001	1995–2008	1.4 (1.3–1.5)	< 0.001	2008–2019	0.3 (0.2–0.4)	< 0.001			
Canada	1990–1998	1.1 (0.6–1.6)	< 0.001	1998–2006	4.4 (3.7–5)	< 0.001	2006–2015	− 1.6 (− 2.1–1.2)	< 0.001	2015–2019	0.3 (− 1.2–1.8)	0.675
United States of America	1990–1995	1.9 (1.6–2.3)	< 0.001	1995–2003	0 (− 0.2–0.2)	0.89	2003–2016	− 0.9 (− 1–0.8)	< 0.001	2016–2019	0 (− 0.7–0.8)	0.909
African Region	1990–2003	0.9 (0.8–0.9)	< 0.001	2003–2009	1.6 (1.3–1.9)	< 0.001	2009–2013	2.4 (1.8–3.1)	< 0.001	2013–2019	1.5 (1.3–1.8)	< 0.001
Region of the Americas	1990–1995	0.9 (0.6–1.3)	< 0.001	1995–1999	− 1.5 (− 2.3–0.7)	0.001	1999–2003	0.2 (− 0.6–1)	0.632	2003–2019	− 0.5 (− 0.5–0.4)	< 0.001
South-East Asia Region	1990–1998	2.5 (2.3–2.8)	< 0.001	1998–2005	− 0.1 (− 0.4–0.3)	0.776	2005–2012	0.9 (0.5–1.3)	< 0.001	2012–2019	2.2 (1.9–2.5)	< 0.001
European Region	1990–1993	2.9 (1.9–3.9)	< 0.001	1993–2008	1.3 (1.2–1.4)	< 0.001	2008–2019	0.1 (− 0.1–0.2)	0.347			
Eastern Mediterranean Region	1990–1996	2.3 (2–2.6)	< 0.001	1996–1999	1.5 (− 0.2–3.3)	0.09	1999–2019	2.6 (2.5–2.6)	< 0.001			
Western Pacific Region	1990–1997	1.2 (0.8–1.7)	< 0.001	1997–2004	4.4 (3.8–5)	< 0.001	2004–2008	2.5 (0.9–4.3)	0.005	2008–2019	0.2 (0–0.4)	0.118

EAPC indicates estimated annual percentage change, with 95% confidence intervals in brackets. *Significantly different from 0 (P < 0.05). AAPC (Average annual percentage change) indicates summary measure of the trend over 1990–2019.

**Figure 3:**
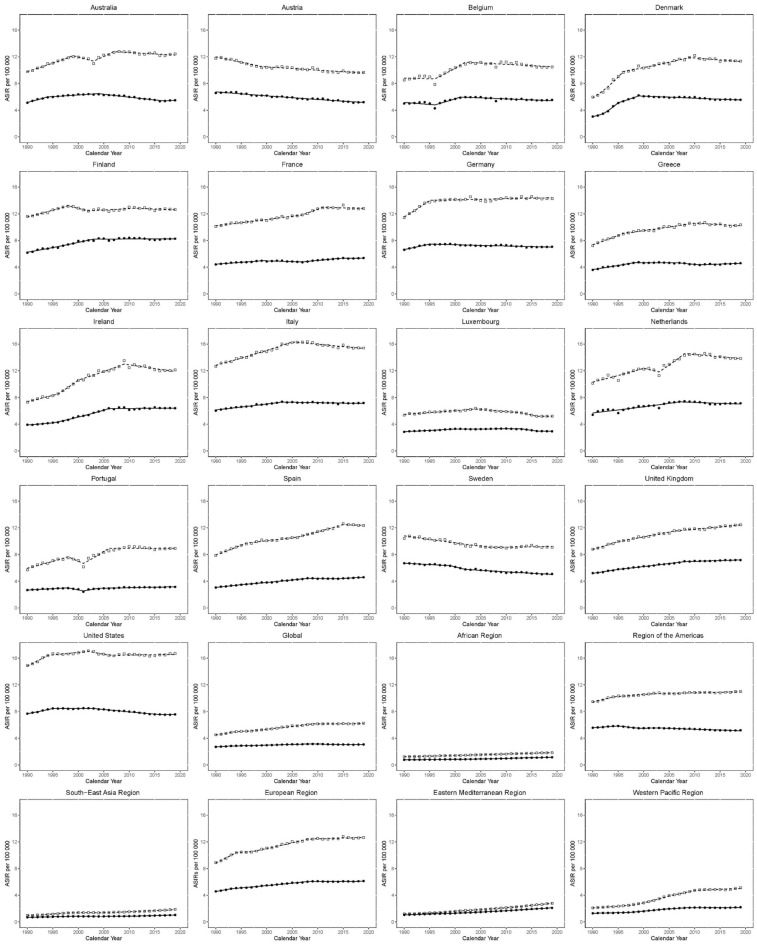
Trends in age-standardized incidence rates (ASIR) per 100,000 for KC in EU15 + countries and WHO regions between 1990 and 2019. Open squares indicate males; and filled circles, females.

Global ASIR for males increased between 1990 and 1994 (EAPC 2.6 [2.1–3.2]), but from 1994 to 1997, the change was not statistically significant (EAPC: 0.8 [− 1.0 to 2.5]). Between 1997 and 2009, the ASIR increased again (EAPC; 1.6 [1.4–1.7]), and since 2009, the trajectory in ASIR has been mostly flat (EAPC 0.1 [− 0.1 to 0.2]). Amongst EU15 + countries, recent trends have been mostly flat or negative. Most recently, the greatest increase has been observed in the UK between 2006 and 2019 (EAPC: 0.6 [0.5–0.7]). The greatest decrease has been observed in Norway between 2011 and 2019 (EAPC: − 2.1 [− 2.6 to − 1.5]).

The rate of increase in ASIR has been variable amongst the WHO regions. Between 2011 and 2019, the Eastern Mediterranean region observed the greatest increase in ASIR (EAPC: 3.1 [2.9–3.3]). Conversely, the Americas have seen the slowest rate of increase between 2002 and 2019 (EAPC: 0.1 [0.1–0.2]).

Global ASIR for females increased between 1990 and 1993 (EAPC; 1.5 [0.8–2.3)], the rate of growth slowed between 1993 and 2009 (EAPC; 0.7 [0.6–0.7]). Between 2009 and 2014, ASIR decreased (EAPC; − 0.7 [− 1.1 to − 0.2]), while the curve has been flat between 2014 and 2019 (EAPC; 0.0 [− 0.3 to 0.4]). As with males, recent ASIR trends in females have mostly been flat or negative. The observed increase in Australia between 2017 and 2019 (EAPC: 1.1 [− 2.6 to 4.9]) was statistically non-significant. The greatest decrease has been observed in Austria between 1990 and 2019 (EAPC: − 0.9 [− 1 to − 0.9]).

Recent ASIR trends in WHO regions for females have mostly been positive. The Eastern Mediterranean region has seen the greatest increase between 1999 and 2019 (EAPC: 2.6 [2.5–2.6]). The Americas are the only region to see a fall recently, between 2003 and 2019 (EAPC: − 0.5 [− 0.5 to − 0.4]).

### Trends in KC ASMR, 1990–2019

Across the entire study period, there was a rise in ASMR globally for males (+ 19.4%) and a fall for females (− 2.3%). ASMR mainly increased across most WHO regions. The Eastern Mediterranean region had the greatest increase across the study period for males and females, + 87.8% and + 43.0%, respectively. The Americas had the smallest increase for males (+ 8.3%) and was the only region to see a decrease in females (− 16.4%). Recently, the Eastern Mediterranean region observed the greatest increase between 2011 and 2019 (EAPC: 2.3 [2.1–2.4]). The Western Pacific is the only region to observe a decrease recently, between 2011 and 2019 (EAPC: − 0.4 [− 0.7 to − 0.2]).

Among EU 15 + countries, Denmark had the greatest increase in ASMR across the study period for males and females, + 41.7% and 37.7%, respectively. Conversely, Austria had the greatest decrease for males and females, − 33.8% and − 35.8% respectively.

Tables 1 and 2 and, Figs. 1, 2 depict gender-specific trends in KC ASMR across EU 15 + countries and 6 WHO regions.

#### The Joinpoint analyses for ASMR in males and females are shown in Table 4 and Fig. 4

**Table 4:** Joinpoint analysis for KC age-standardized mortality rates in EU15 + countries and WHO regions for years 1990–2019 in males (A), females (B) and both sexes (C).

(A)												
Country/Region	Trend 1			Trend 2			Trend 3			Trend 4		
	Years	EAPC (95% CI)	“P-value”	Years	EAPC (95% CI)	“P-value”	Years	EAPC (95% CI)	“P-value”	Years	EAPC (95% CI)	“P-value”
Global	1990–1993	1.9 (1–2.8)	< 0.001	1993–2009	0.9 (0.8–0.9)	< 0.001	2009–2019	− 0.2 (− 0.3–0)	0.018			
Australia	1990–1999	0.6 (0.3–0.8)	< 0.001	1999–2003	− 3.1 (− 4.4–1.8)	< 0.001	2003–2006	2.9 (0.1–5.6)	0.041	2006–2019	− 0.5 (− 0.6–0.3)	< 0.001
Austria	1990–2000	− 2.8 (− 2.9–2.6)	< 0.001	2000–2003	0.4 (− 1.9–2.8)	0.721	2003–2012	− 1.4 (− 1.6–1.1)	< 0.001	2012–2019	− 0.4 (− 0.8–0.1)	0.008
Belgium	1990–1993	1.9 (− 1.5–5.4)	0.261	1993–1996	− 4.5 (− 10.7–2.1)	0.168	1996–1999	6.9 (0–14.4)	0.051	1999–2019	− 0.6 (− 0.8–0.4)	< 0.001
Denmark	1990–1996	8.2 (7.4–9.1)	< 0.001	1996–2009	0 (− 0.3–0.3)	0.881	2009–2019	− 1.1 (− 1.5–0.8)	< 0.001			
Finland	1990–1998	0.4 (0.2–0.6)	< 0.001	1998–2008	− 1.6 (− 1.8–1.5)	< 0.001	2008–2011	1.2 (− 0.2–2.6)	0.098	2011–2019	− 0.6 (− 0.8–0.5)	< 0.001
France	1990–2007	− 0.5 (− 0.5–0.4)	< 0.001	2007–2011	1.7 (0.7–2.7)	0.001	2011–2019	− 0.5 (− 0.7–0.3)	< 0.001			
Germany	1990–1996	2.3 (1.9–2.7)	< 0.001	1996–2007	− 1.3 (− 1.5–1.1)	< 0.001	2007–2013	1.2 (0.7–1.8)	< 0.001	2013–2019	− 0.3 (− 0.7–0.1)	0.147
Greece	1990–1992	4.1 (2.1–6.1)	< 0.001	1992–1998	1.8 (1.3–2.2)	< 0.001	1998–2019	− 0.3 (− 0.3–0.2)	< 0.001			
Ireland	1990–1996	1.7 (1.1–2.2)	< 0.001	1996–2001	3.5 (2.4–4.6)	< 0.001	2001–2009	0.3 (− 0.1–0.7)	0.172	2009–2019	− 1.3 (− 1.5–1)	< 0.001
Italy	1990–1994	0.8 (0.2–1.3)	0.009	1994–2001	− 0.1 (− 0.4–0.2)	0.614	2001–2005	0.6 (− 0.2–1.5)	0.14	2005–2019	− 0.6 (− 0.7–0.5)	< 0.001
Luxembourg	1990–2004	− 0.5 (− 0.6–0.4)	< 0.001	2004–2013	− 1.4 (− 1.7–1.2)	< 0.001	2013–2016	− 3.3 (− 5.6–1)	0.008	2016–2019	0.1 (− 1.1–1.3)	0.915
Netherlands	1990–2000	1 (0.6–1.5)	< 0.001	2000–2003	− 3.5 (− 9–2.2)	0.209	2003–2006	5.8 (− 0.1–12.2)	0.055	2006–2019	− 0.6 (− 0.9–0.3)	0.001
Norway	1990–2012	0.6 (0.5–0.7)	< 0.001	2012–2019	− 2.2 (− 2.6–1.8)	< 0.001						
Portugal	1990–1998	1.3 (0.7–2)	0.001	1998–2001	− 6.8 (− 12.3–0.8)	0.028	2001–2004	7 (0.6–13.8)	0.033	2004–2019	− 0.1 (− 0.3–0.2)	0.571
Spain	1990–1995	1.9 (1.6–2.2)	< 0.001	1995–2005	0.4 (0.2–0.5)	< 0.001	2005–2015	1.6 (1.5–1.7)	< 0.001	2015–2019	− 0.6 (− 1–0.2)	0.01
Sweden	1990–1998	− 0.8 (− 1.1–0.6)	< 0.001	1998–2001	− 2.6 (− 4.8–0.3)	0.029	2001–2008	− 1.5 (− 1.9–1.1)	< 0.001	2008–2019	− 0.1 (− 0.3–0)	0.078
United Kingdom	1990–1995	1.3 (0.9–1.7)	< 0.001	1995–2006	0.3 (0.1–0.4)	< 0.001	2006–2010	− 0.5 (− 1.3–0.3)	0.232	2010–2019	0.3 (0.2–0.5)	< 0.001
Canada	1990–2001	0.4 (0.2–0.6)	0.001	2001–2004	5.7 (2.3–9.3)	0.002	2004–2008	1.2 (− 0.5–2.8)	0.167	2008–2019	− 0.4 (− 0.6–0.2)	0.001
United States of America	1990–1995	1 (0.5–1.4)	< 0.001	1995–2014	− 0.5 (− 0.6–0.4)	< 0.001	2014–2019	0.7 (0.2–1.1)	0.006			
African Region	1990–1998	1.5 (1.3–1.6)	< 0.001	1998–2019	1 (1–1.1)	< 0.001						
Region of the Americas	1990–1994	0.8 (0.4–1.2)	< 0.001	1994–2002	0.3 (0.1–0.4)	0.002	2002–2015	0.1 (0–0.1)	0.063	2015–2019	0.5 (0.2–0.9)	0.005
South-East Asia Region	1990–1993	2.3 (0.7–4)	0.007	1993–1998	4.4 (3.4–5.5)	< 0.001	1998–2011	0.4 (0.2–0.6)	< 0.001	2011–2019	1.8 (1.5–2.2)	< 0.001
European Region	1990–1994	3.5 (2.8–4.2)	< 0.001	1994–1997	− 0.4 (− 2.4–1.7)	0.686	1997–2005	0.8 (0.5–1.1)	< 0.001	2005–2019	0 (− 0.1–0.1)	0.55
Eastern Mediterranean Region	1990–1996	1.9 (1.6–2.1)	< 0.001	1996–2001	3.2 (2.7–3.7)	< 0.001	2001–2011	2 (1.8–2.1)	< 0.001	2011–2019	2.3 (2.1–2.4)	< 0.001
Western Pacific Region	1990–1998	1.8 (1.5–2)	< 0.001	1998–2005	4.8 (4.4–5.3)	< 0.001	2005–2011	2 (1.4–2.6)	< 0.001	2011–2019	− 0.4 (− 0.7–0.2)	0.003

(B)												
Country/Region	Trend 1			Trend 2			Trend 3			Trend 4		
	Years	EAPC (95% CI)	“P-value”	Years	EAPC (95% CI)	“P-value”	Years	EAPC (95% CI)	“P-value”	Years	EAPC (95% CI)	“P-value”
Global	1990–1994	0.9 (0.5–1.2)	< 0.001	1994–1997	− 0.4 (− 1.5–0.6)	0.409	1997–2006	0.3 (0.1–0.4)	< 0.001	2006–2019	− 0.6 (− 0.6–0.5)	< 0.001
Australia	1990–1993	2.6 (1.8–3.4)	< 0.001	1993–2008	− 1 (− 1.1–0.9)	< 0.001	2008–2016	− 2.1 (− 2.3–1.9)	< 0.001	2016–2019	0.8 (0–1.6)	0.046
Austria	1990–1992	− 0.3 (− 2.8–2.2)	0.793	1992–2000	− 2.3 (− 2.6–2)	< 0.001	2000–2017	− 1.5 (− 1.6–1.4)	< 0.001	2017–2019	0.2 (− 2.2–2.7)	0.851
Belgium	1990–1993	1.3 (− 2.8–5.7)	0.513	1993–1996	− 6 (− 13.6–2.2)	0.14	1996–1999	7.1 (− 1.5–16.5)	0.104	1999–2019	− 1.1 (− 1.3–0.8)	< 0.001
Denmark	1990–1992	6.4 (1.4–11.6)	0.014	1992–1995	12.8 (7.5–18.3)	< 0.001	1995–1998	4.7 (− 0.2–9.8)	0.059	1998–2019	− 1.6 (− 1.7–1.5)	< 0.001
Finland	1990–2003	0.9 (0.8–1)	< 0.001	2003–2006	− 1.6 (− 4.2–1.1)	0.229	2006–2019	− 0.3 (− 0.5–0.2)	< 0.001			
France	1990–1999	− 0.2 (− 0.3–0)	0.017	1999–2007	− 1.8 (− 2–1.6)	< 0.001	2007–2012	1.2 (0.8–1.7)	< 0.001	2012–2019	0 (− 0.2–0.2)	0.934
Germany	1990–1994	1.4 (1–1.8)	< 0.001	1994–1998	0.1 (− 0.5–0.7)	0.638	1998–2005	− 1.7 (− 1.9–1.5)	< 0.001	2005–2019	0 (− 0.1–0)	0.227
Greece	1990–1999	2.3 (2.1–2.5)	< 0.001	1999–2008	− 1.6 (− 1.8–1.3)	< 0.001	2008–2011	− 3.5 (− 5.9–1)	0.008	2011–2019	0.5 (0.2–0.8)	0.001
Ireland	1990–1994	0.8 (− 0.2–1.7)	0.1	1994–2006	2 (1.8–2.2)	< 0.001	2006–2011	− 1.3 (− 2.2–0.4)	0.007	2011–2019	− 0.2 (− 0.5–0.1)	0.265
Italy	1990–1992	1 (− 0.1–2.1)	0.071	1992–2003	− 0.1 (− 0.2–0)	0.091	2003–2014	− 0.4 (− 0.5–0.3)	< 0.001	2014–2019	0.1 (− 0.2–0.3)	0.445
Luxembourg	1990–2000	− 0.3 (− 0.4–0.1)	0.005	2000–2019	− 1.1 (− 1.2–1.1)	< 0.001						
Netherlands	1990–2007	0.4 (0.1–0.8)	0.016	2007–2019	− 0.8 (− 1.4–0.2)	0.011						
Norway	1990–2001	1.6 (1.4–1.7)	< 0.001	2001–2012	0 (− 0.1–0.2)	0.598	2012–2017	− 2.4 (− 3.1–1.7)	< 0.001	2017–2019	0.7 (− 1.6–2.9)	0.539
Portugal	1990–1998	0 (− 0.3–0.4)	0.817	1998–2001	− 6.4 (− 9.3–3.5)	< 0.001	2001–2005	2.5 (0.9–4.1)	0.004	2005–2019	− 0.1 (− 0.3–0)	0.058
Spain	1990–2008	0.5 (0.5–0.6)	< 0.001	2008–2015	− 0.2 (− 0.5–0.1)	0.167	2015–2019	0.8 (0.2–1.4)	0.009			
Sweden	1990–1998	− 0.9 (− 1–0.7)	< 0.001	1998–2002	− 2.8 (− 3.5–2)	< 0.001	2002–2009	− 1.7 (− 2–1.5)	< 0.001	2009–2019	− 0.6 (− 0.7–0.5)	< 0.001
United Kingdom	1990–1998	1.1 (1–1.2)	< 0.001	1998–2008	0.5 (0.4–0.5)	< 0.001	2008–2019	0 (− 0.1–0.1)	0.708			
Canada	1990–1998	0 (− 0.5–0.6)	0.883	1998–2006	4.1 (3.5–4.8)	< 0.001	2006–2016	− 1.9 (− 2.4–1.5)	< 0.001	2016–2019	0.3 (− 2–2.7)	0.764
United States of America	1990–1995	0.8 (0.5–1)	< 0.001	1995–2003	− 0.5 (− 0.6–0.4)	< 0.001	2003–2017	− 1 (− 1–0.9)	< 0.001	2017–2019	1 (0–2)	0.056
African Region	1990–2004	0.5 (0.5–0.6)	< 0.001	2004–2008	1.2 (0.5–1.9)	0.002	2008–2014	1.9 (1.6–2.2)	< 0.001	2014–2019	1 (0.7–1.3)	< 0.001
Region of the Americas	1990–1994	− 0.1 (− 0.4–0.2)	0.585	1994–1999	− 2.1 (− 2.4–1.7)	< 0.001	1999–2017	− 0.4 (− 0.5–0.4)	< 0.001	2017–2019	0.3 (− 0.7–1.3)	0.586
South-East Asia Region	1990–1999	1.8 (1.6–2)	< 0.001	1999–2004	− 0.5 (− 1.2–0.2)	0.13	2004–2012	0 (− 0.3–0.3)	0.81	2012–2019	1.5 (1.2–1.8)	< 0.001
European Region	1990–1994	2.2 (1.7–2.7)	< 0.001	1994–2005	0.6 (0.5–0.8)	< 0.001	2005–2019	− 0.2 (− 0.3–0.2)	< 0.001			
Eastern Mediterranean Region	1990–1995	1.3 (1.1–1.6)	< 0.001	1995–2000	0.3 (0–0.7)	0.077	2000–2016	1.6 (1.5–1.6)	< 0.001	2016–2019	0.9 (0.3–1.4)	0.006
Western Pacific Region	1990–1997	0.5 (0.2–0.7)	0.001	1997–2006	2.7 (2.4–2.9)	< 0.001	2006–2017	− 0.7 (− 0.9–0.6)	< 0.001	2017–2019	0.7 (− 1.2–2.7)	0.444

EAPC indicates estimated annual percentage change, with 95% confidence intervals in brackets.

*Significantly different from 0 (P < 0.05). AAPC (Average annual percentage change) indicates summary measure of the trend over 1990–2019.

**Figure 4:**
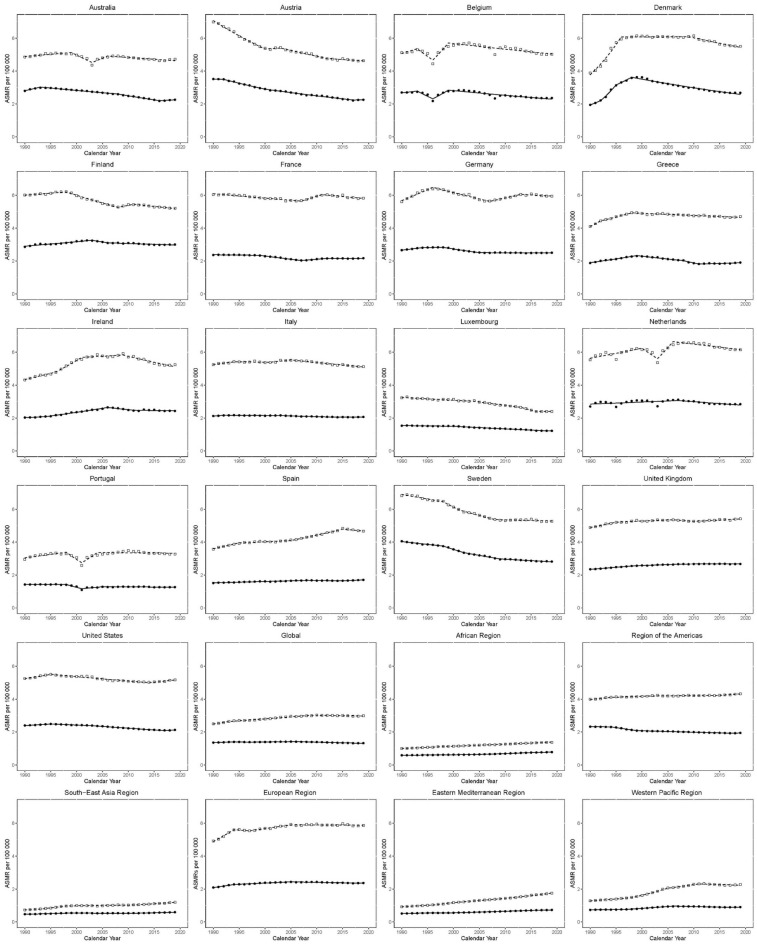
Trends in age-standardized mortality rates (ASMR) per 100,000 for KC in EU15 + countries and WHO regions between 1990 and 2019. Open squares indicate males; and filled circles, females.

Global ASMR for males increased between 1990 and 1993 (EAPC: 1.9 [1–2.8]). Between 1993 and 2009, the rate of increase slowed (EAPC: 0.9 [0.8–0.9]). Between 2009 and 2019, there has been a non-significant decrease in ASMR (EAPC: − 0.2 [− 0.3 to 0]).

Amongst EU 15 + countries, recent trends have mostly been negative, except in the USA, in which there has been a recent growth in ASMR between 2014 and 2019 (EAPC 0.7 [0.2–1.1]). Recently, Norway has observed the greatest decrease between 2012 and 2019 (EAPC: − 2.2 [− 2.6 to − 1.8]). WHO regions have mostly seen an increase in ASMR recently.

Global, ASMR for females increased between 1990 and 1994 (EAPC: 0.9 [0.5–1.2]). The trend reversed but was statistically non-significant between 1994 and 1997 (EAPC: − 0.4 [− 1.5 to 0.6]). Again, there was an increase in ASMR between 1997 and 2006 (EAPC: 0.3 [0.1–0.4]). The trend between 2006 and 2019 showed an improvement in mortality (EAPC: − 0.6 [− 0.6 to − 0.5]). The South-East Asian region has seen the largest increase between 2012 and 2019 (EAPC: 1.5 [1.2–1.8]). Europe was the only region with negative growth recently, between 2005 and 2019 (EAPC: − 0.2 [− 0.3 to − 0.2]).

Amongst EU 15 + countries, recent trends have been mixed. The greatest increase but statistically non-significant was observed in the USA between 2017 and 2019 (EAPC: 1 [0–2]). The greatest decrease was observed in Denmark between 1998 and 2019 (EAPC: − 1.6 [− 1.7 to − 1.5]). WHO regions have mostly seen an increase in ASMR recently.

### Trends in KC disability-adjusted life-years, 1990–2019

Across the 29-year study period, there was a rise in DALYs globally for males (+ 13.1%) and a fall for females (− 9.4%). Similar trends were seen across all six regions as well. The Eastern Mediterranean region saw the greatest increase for males (+ 76.6%), whereas the Americas saw the smallest rise (+ 2.1%). Apart from the Americas (− 23.5%), all regions saw a rise in DALYs for females, led by the Eastern Mediterranean region (+ 36.3%).

In the EU15 + countries, Denmark saw the greatest rise in DALYs for both males and females, + 38.6% and + 30.2 respectively. Austria saw the greatest decrease in DALYs across the study period for males and females, − 39.5% and − 41.2%, respectively. Figures 1 and 2 depict gender-specific trends in KC DALYs across EU 15 + countries and 6 WHO regions.

Tables 1 and 2 and, Figs. 1 and 2 depict gender-specific trends in KC DALY across EU 15 + countries and 6 WHO regions.

#### The Joinpoint analyses for DALYs in males and females are shown in Table 5 and Fig. 5

**Table 5:** Joinpoint analysis for KC age-standardized DALYs in EU15 + countries and WHO regions for years 1990–2019 for both sexes.

(A)												
Country/Region	Trend 1			Trend 2			Trend 3			Trend 4		
	Years	EAPC (95% CI)	“P-value”	Years	EAPC (95% CI)	“P-value”	Years	EAPC (95% CI)	“P-value”	Years	EAPC (95% CI)	“P-value”
Global	1990–1994	1.8 (1.3–2.2)	< 0.001	1994–1998	− 0.1 (− 0.9–0.6)	0.736	1998–2005	1.2 (0.9–1.4)	< 0.001	2005–2019	− 0.2 (− 0.2–0.1)	< 0.001
Australia	1990–1999	0.4 (0.2–0.6)	0.001	1999–2003	− 3 (− 4.1–1.9)	< 0.001	2003–2007	1.9 (0.8–3.1)	0.002	2007–2019	− 0.6 (− 0.7–0.4)	< 0.001
Austria	1990–1999	− 3 (− 3.3–2.8)	< 0.001	1999–2014	− 1.4 (-− 1.5–1.3)	< 0.001	2014–2019	− 0.5 (− 1.1–0)	0.07			
Belgium	1990–1996	− 1.6 (− 2.5–0.7)	0.001	1996–2001	3.4 (1.7–5.2)	0.001	2001–2019	− 1.1 (− 1.3–0.9)	< 0.001			
Denmark	1990–1996	8.4 (7.5–9.3)	< 0.001	1996–2009	− 0.1 (− 0.4–0.2)	0.352	2009–2019	− 1.2 (− 1.6–0.9)	< 0.001			
Finland	1990–1998	0.1 (− 0.2–0.4)	0.555	1998–2001	− 2.8 (− 5.3–0.4)	0.027	2001–2006	− 1.4 (− 2.2–0.6)	0.002	2006–2019	− 0.7 (− 0.9–0.6)	< 0.001
France	1990–1993	0 (− 0.9–0.9)	0.927	1993–2007	− 0.7 (− 0.8–0.6)	< 0.001	2007–2011	1.2 (0.3–2.2)	0.01	2011–2019	− 0.6 (− 0.8–0.4)	< 0.001
Germany	1990–1995	1.9 (1.5–2.3)	< 0.001	1995–2003	− 1.2 (− 1.5–1)	< 0.001	2003–2006	− 2.2 (− 3.8–0.6)	0.009	2006–2019	− 0.1 (− 0.2–0)	0.031
Greece	1990–1992	3.9 (1.4–6.3)	0.003	1992–1997	1.2 (0.5–2)	0.002	1997–2019	− 0.1 (− 0.1–0)	0.047			
Ireland	1990–1996	1 (0.3–1.6)	0.007	1996–2000	3.8 (1.8–5.9)	0.001	2000–2009	0.3 (− 0.1–0.8)	0.124	2009–2019	− 1.5 (− 1.8–1.1)	< 0.001
Italy	1990–2007	− 0.3 (− 0.4–0.3)	< 0.001	2007–2013	− 1.2 (− 1.6–0.7)	< 0.001	2013–2019	− 0.4 (− 0.7–0.1)	0.017			
Luxembourg	1990–2004	− 0.8 (− 0.9–0.7)	< 0.001	2004–2013	− 1.8 (− 2.1–1.6)	< 0.001	2013–2016	− 3 (− 5.2–0.8)	0.011	2016–2019	0 (− 1.1–1.2)	0.949
Netherlands	1990–2000	0.7 (0.3–1.2)	0.003	2000–2003	− 3.5 (− 8.8–2.2)	0.207	2003–2007	3.4 (0.5–6.4)	0.023	2007–2019	− 0.9 (− 1.2–0.5)	< 0.001
Norway	1990–2013	0.4 (0.4–0.5)	< 0.001	2013–2017	− 3.8 (− 5.3–2.3)	< 0.001	2017–2019	0.3 (− 2.8–3.4)	0.858			
Portugal	1990–1998	0.8 (0.3–1.4)	0.004	1998–2001	− 6.5 (− 11–1.7)	0.011	2001–2007	3.2 (2–4.3)	< 0.001	2007–2019	− 0.8 (− 1.1–0.6)	< 0.001
Spain	1990–1996	1.5 (1.2–1.8)	< 0.001	1996–2006	− 0.1 (− 0.2–0.1)	0.364	2006–2015	1.3 (1.1–1.4)	< 0.001	2015–2019	− 0.4 (− 0.9–0.2)	0.152
Sweden	1990–1998	− 1.4 (− 1.7–1.1)	< 0.001	1998–2002	− 2.9 (− 4.2–1.6)	< 0.001	2002–2009	− 1.2 (− 1.7–0.8)	< 0.001	2009–2019	− 0.4 (− 0.6–0.2)	0.001
United Kingdom	1990–1996	1.1 (0.8–1.3)	< 0.001	1996–2007	-0.2 (-0.4–0.1)	< 0.001	2007–2012	-0.8 (-1.3–0.3)	0.002	2012–2019	0.3 (0.1–0.5)	0.01
Canada	1990–2001	0 (− 0.2–0.2)	0.814	2001–2004	5.1 (2.2–8.2)	0.002	2004–2008	1 (− 0.4–2.5)	0.153	2008–2019	− 0.5 (− 0.7–0.3)	< 0.001
United States of America	1990–1995	0.8 (0.4–1.3)	0.001	1995–2013	− 0.9 (− 0.9–0.8)	< 0.001	2013–2019	0.5 (0.1–0.8)	0.01			
African Region	1990–2006	1 (0.9–1)	< 0.001	2006–2009	0.1 (− 0.9–1.1)	0.882	2009–2017	0.8 (0.7–1)	< 0.001	2017–2019	− 0.1 (− 1.1–0.9)	0.789
Region of the Americas	1990–1994	0.6 (0.2–1)	0.006	1994–2015	− 0.1 (− 0.1–0.1)	< 0.001	2015–2019	0.5 (0.1–0.9)	0.018			
South-East Asia Region	1990–1999	3.2 (2.9–3.5)	< 0.001	1999–2004	− 0.3 (− 1.2–0.7)	0.551	2004–2014	1 (0.7–1.2)	< 0.001	2014–2019	1.8 (1.2–2.5)	< 0.001
European Region	1990–1994	3.7 (2.9–4.4)	< 0.001	1994–1997	− 1.1 (− 3.3–1.3)	0.349	1997–2005	0.6 (0.2–0.9)	0.001	2005–2019	− 0.4 (− 0.5–0.3)	< 0.001
Eastern Mediterranean Region	1990–1996	1.6 (1.3–1.9)	< 0.001	1996–2000	2.9 (2.1–3.8)	< 0.001	2000–2019	1.9 (1.9–2)	< 0.001			
Western Pacific Region	1990–1998	0.9 (0.5–1.2)	< 0.001	1998–2005	5.1 (4.6–5.7)	< 0.001	2005–2011	1.5 (0.8–2.2)	< 0.001	2011–2019	− 0.5 (− 0.8–0.1)	0.011

(B)												
Country/Region	Trend 1			Trend 2			Trend 3			Trend 4		
	Years	EAPC (95% CI)	“P-value”	Years	EAPC (95% CI)	“P-value”	Years	EAPC (95% CI)	“P-value”	Years	EAPC (95% CI)	“P-value”
Global	1990–1994	0.4 (− 0.1–0.9)	0.106	1994–1997	− 1.1 (− 2.7–0.5)	0.164	1997–2005	− 0.1 (− 0.3–0.2)	0.599	2005–2019	− 0.7 (− 0.7–0.6)	< 0.001
Australia	1990–1993	2 (1.3–2.6)	< 0.001	1993–2011	− 1.3 (− 1.4–1.3)	< 0.001	2011–2016	− 2.4 (− 2.8–2)	< 0.001	2016–2019	0.9 (0.3–1.6)	0.009
Austria	1990–1992	− 0.4 (− 3.6–2.9)	0.8	1992–2001	− 2.7 (− 3–2.4)	< 0.001	2001–2017	− 1.7 (− 1.9–1.6)	< 0.001	2017–2019	0.1 (− 3–3.4)	0.925
Belgium	1990–1993	0.4 (− 3.3–4.3)	0.817	1993–1996	− 6 (− 12.9–1.4)	0.105	1996–1999	6.5 (− 1.3–15)	0.1	1999–2019	− 1.3 (− 1.5–1.1)	< 0.001
Denmark	1990–1996	9.8 (8.9–10.8)	< 0.001	1996–1999	2.4 (− 2.9–7.9)	0.361	1999–2014	− 2.1 (− 2.3–1.8)	< 0.001	2014–2019	− 0.5 (− 1.7–0.6)	0.345
Finland	1990–2002	0.7 (0.5–0.9)	< 0.001	2002–2019	− 0.8 (− 0.9–0.7)	< 0.001						
France	1990–2000	− 0.5 (− 0.6–0.4)	< 0.001	2000–2007	− 2.4 (− 2.6–2.2)	< 0.001	2007–2011	0.9 (0.2–1.5)	0.011	2011–2019	0.2 (0.1–0.3)	0.006
Germany	1990–1997	− 0.1 (− 0.3–0.1)	0.192	1997–2005	− 2.2 (− 2.3–2)	< 0.001	2005–2015	− 0.8 (− 0.9–0.6)	< 0.001	2015–2019	0.2 (− 0.2–0.7)	0.302
Greece	1990–1999	1.5 (1.2–1.7)	< 0.001	1999–2011	− 1.7 (− 1.9–1.5)	< 0.001	2011–2019	0.6 (0.3–0.9)	0.001			
Ireland	1990–1994	− 0.3 (− 1.2–0.6)	0.473	1994–2006	1.8 (1.6–2)	< 0.001	2006–2010	− 1.9 (− 3.3–0.5)	0.01	2010–2019	− 0.4 (− 0.6–0.1)	0.005
Italy	1990–1994	0.1 (− 0.4–0.6)	0.586	1994–2008	− 0.9 (− 1–0.8)	< 0.001	2008–2015	− 0.6 (− 0.8–0.3)	< 0.001	2015–2019	0.2 (− 0.3–0.7)	0.397
Luxembourg	1990–2001	− 0.6 (− 0.8–0.5)	< 0.001	2001–2004	− 1.8 (− 4.1–0.6)	0.134	2004–2019	− 1.1 (− 1.2–1)	< 0.001			
Netherlands	1990–2006	0.1 (-0.3–0.4)	0.738	2006–2019	− 1.1 (− 1.6–0.6)	< 0.001						
Norway	1990–2001	1.2 (1–1.3)	< 0.001	2001–2012	− 0.4 (− 0.6–0.3)	< 0.001	2012–2017	− 2.9 (− 3.6–2.2)	< 0.001	2017–2019	0.9 (− 1.3–3.3)	0.393
Portugal	1990–1998	− 0.8 (− 1.1–0.5)	< 0.001	1998–2001	− 5.7 (− 8.5–2.8)	0.001	2001–2019	− 0.3 (− 0.4–0.2)	< 0.001			
Spain	1990–2008	0.2 (0.2–0.3)	< 0.001	2008–2014	− 0.7 (− 1.2–0.3)	0.004	2014–2019	0.7 (0.3–1.2)	0.003			
Sweden	1990–1998	− 1.5 (− 1.7–1.3)	< 0.001	1998–2002	− 3.2 (− 4.1–2.2)	< 0.001	2002–2009	− 2 (− 2.3–1.6)	< 0.001	2009–2019	− 1 (− 1.2–0.9)	< 0.001
United Kingdom	1990–1997	0.8 (0.7–1)	< 0.001	1997–2008	0 (− 0.1–0.1)	0.759	2008–2014	− 0.7 (− 0.9–0.5)	< 0.001	2014–2019	0 (− 0.3–0.2)	0.813
Canada	1990–1998	− 0.2 (− 0.7–0.3)	0.357	1998–2006	3.3 (2.7–3.9)	< 0.001	2006–2014	− 2.4 (− 3–1.8)	< 0.001	2014–2019	− 0.3 (− 1.3–0.7)	0.517
United States of America	1990–1995	0.5 (0.2–0.7)	0.001	1995–2003	− 1 (− 1.2–0.9)	< 0.001	2003–2014	− 1.4 (− 1.5–1.4)	< 0.001	2014–2019	− 0.1 (− 0.3–0.2)	0.464
African Region	1990–2004	0.3 (0.3–0.4)	< 0.001	2004–2009	1 (0.6–1.5)	< 0.001	2009–2014	1.8 (1.4–2.3)	< 0.001	2014–2019	0.8 (0.4–1.1)	< 0.001
Region of the Americas	1990–1995	− 0.8 (− 1–0.7)	< 0.001	1995–1999	− 3.2 (− 3.6–2.8)	< 0.001	1999–2014	− 0.6 (− 0.7–0.6)	< 0.001	2014–2019	− 0.1 (− 0.3–0.1)	0.351
South-East Asia Region	1990–1999	1.7 (1.5–1.9)	< 0.001	1999–2004	− 0.7 (− 1.3–0)	0.049	2004–2012	0.2 (− 0.1–0.5)	0.184	2012–2019	1.4 (1.1–1.7)	< 0.001
European Region	1990–1994	1.8 (1.3–2.4)	< 0.001	1994–2005	0.2 (0–0.3)	0.011	2005–2017	− 0.7 (− 0.8–0.6)	< 0.001	2017–2019	0.5 (− 1.3–2.2)	0.578
Eastern Mediterranean Region	1990–1994	1.2 (0.8–1.6)	< 0.001	1994–2002	0.6 (0.4–0.7)	< 0.001	2002–2016	1.4 (1.4–1.5)	< 0.001	2016–2019	0.5 (− 0.1–1.1)	0.081
Western Pacific Region	1990–1997	− 0.7 (− 1.1–0.4)	0.001	1997–2005	2.7 (2.3–3.1)	< 0.001	2005–2016	− 1.1 (− 1.3–0.9)	< 0.001	2016–2019	0.4 (− 1–1.8)	0.572

EAPC indicates estimated annual percentage change, with 95% confidence intervals in brackets.

*Significantly different from 0 (P < 0.05). AAPC (Average annual percentage change) indicates summary measure of the trend over 1990–2019.

**Figure 5:**
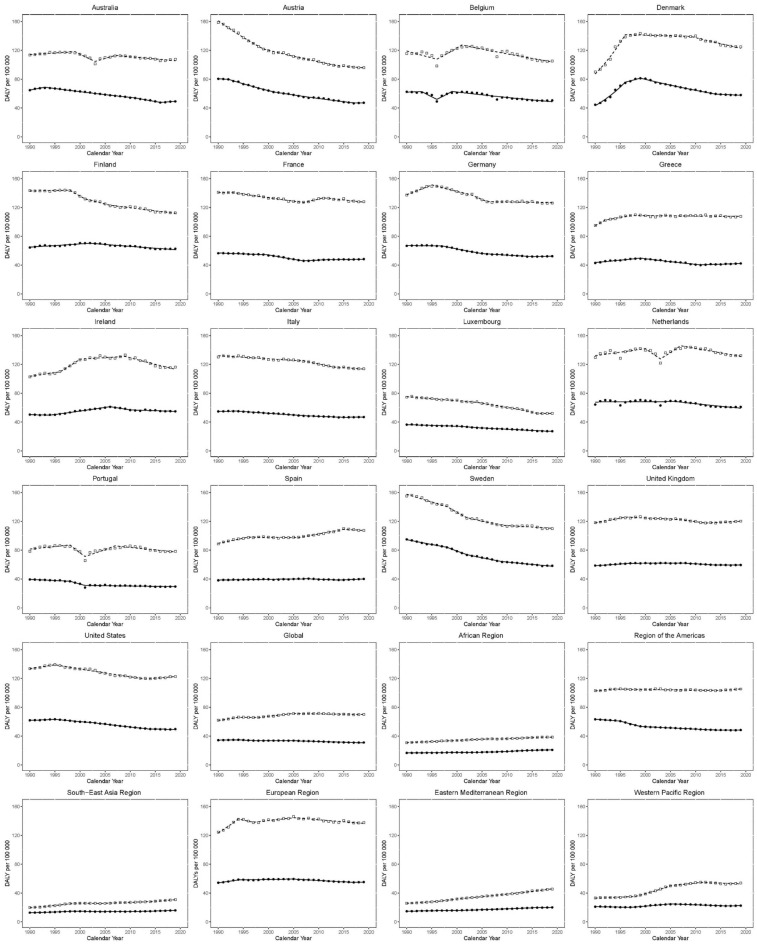
Trends in disability-adjusted life years (DALYs) per 100,000 for KC in EU15 + countries and WHO regions between 1990 and 2019. Open squares indicate males; and filled circles, females.

Globally, DALYs for males were increasing from 1990 to 1994 (EAPC: 1.8 [1.3–2.2]), followed by a slight decrease from 1994 to 1998 (EAPC: − 0.1 [− 0.9 to 1.4]) and an increase between 1998 to 2005 (EAPC: 1.2 [0.9–1.4]). Since 2005, DALYs have plateaued (EAPC: − 0.2 [− 0.2 to 0.1]).

Amongst EU15 + countries, recent trends have mainly shown reductions, with the greatest decrease amongst males in Ireland from 2009 to 2019 (EAPC: − 1.5 [− 1.8 to 1.1)]. The greatest rise recently was seen in the USA from 2013 to 2019 (EAPC: 0.5 [0.1–0.8]).

Trends have been mixed amongst WHO regions recently. The Eastern Mediterranean region has had the greatest increase recently, between 2000 and 2019 (EAPC; 1.9 [1.9–2.0]). Meanwhile, the Western Pacific region had the greatest decrease recently, between 2011 and 2019 (EAPC; − 0.5 [− 0.8 to − 0.1]). Also, trends across WHO regions have mainly been non-significantly positive recently. The greatest recent rise was seen in South-East Asia between 2012 and 2019 (EAPC: 1.4 [1.1–1.7]). The Americas were the only region with a decrease, albeit not significant (EAPC; − 0.1 [− 0.3 to − 0.1]).

Similar DALYs trends were seen globally for females. Between 1990 and 1994, there was a moderate increase (EAPC: 0.4 [− 0.1 to 0.9)]. Since 1994, there has been a non-significant decrease till 2005. Since 2005, there has been a significant decrease (EAPC: − 0.7 [− 0.7 to − 0.6]). Amongst EU15 + countries, recent trends have been mostly negative for males. The most recent significant increase was observed in Australia between 2016 and 2019 (EAPC: 0.9 [0.3–1.6]). The greatest decrease was observed in Belgium between 1999 and 2019 (EAPC: − 1.3 [− 1.5 to − 1.1]).

### Trends in KC MIR, 1990–2019

Across the study period, there was a decrease in MIR globally for both males (− 13.7%) and females (− 13.9%). All EU 15 + countries saw a decrease in MIR across the study period. Portugal saw the greatest decrease in males with a − 29.0% fall, while Ireland saw the greatest decrease in females with a − 26.6% fall. Sweden saw the smallest decrease in males and females with only a − 11.7% and − 8.5% fall, respectively. These trends were reflected across WHO regions, where all regions saw decreases in MIR across the study period. The Western Pacific region saw the greatest decrease in MIR in males with a − 27.3% reduction, while the Western Pacific and Eastern Mediterranean region were equivalent for females with − 27.9% reductions. The Americas saw the smallest decrease in MIR for both males and females over the study period, − 6.9% and − 10.2%, respectively.

## Discussion

In this study, we aimed to analyze the trends of incidence, mortality, mortality to incidence ratio, and DALYs associated with KC among EU15 + countries and 6 WHO regions, using the GBD study data and Joinpoint regression analysis. During a 29-year interval, while incidence and mortality from KC increased in most of the included countries, a drop in MIR was noted in all countries. Trends in DALYs were variable between countries. High-income countries had the highest values of ASIR, ASMR, and DALYs, concurring with previous studies^11,23^. This could be partly attributed to a better performing cancer registry system and a higher prevalence of KC-related risk factors^23^.

We found that the incidence of KC has been increasing in most EU15 + countries, which is congruent with previous reports^11,23^. The rise in incidence is likely in part due to greater detection of early-stage KC on cross-sectional imaging and partly due to the increasing prevalence of smoking, obesity, and hypertension which are among the strongest risk factors of KC^5–7,11,24^. In terms of smoking, an addition of more than 200 million daily smokers was noted between 1980 and 2012, probably contributing to the development of KC worldwide^25,26^. However, recent declines in smoking rates have occurred in developed countries due to anti-smoking campaigns. This trend may be reflected by the decline in EAPC seen in the last decade in the high-income and upper-middle-income countries as per World Bank classification^23^. Similarly, worldwide obesity prevalence had doubled during the past decades, with increasing trends seen especially in developed countries^27,28^. This increase in obesity prevalence parallels our results showing that developed countries had a notable increase in incidence with the highest elevations noted in the World Bank upper-middle-income category for males. Occupational exposures to toxic compounds such as cadmium have also been found to elevate the risk of KC among males, however, to a lesser extent^29^. Even though the contribution ratio of occupational exposure to trichloroethylene to kidney cancer incidence and mortality was relatively weak in the GBD database, protection is mandatory in the organic/chlorinated solvent industry^23^. Recent analysis of risk factor contribution to global cancer burden in 2019 showed that for both sex combined, 33.8% of deaths could be attributed to risk factors analyzed with 19% attributed to High body-mass index, with 33% absolute increase since 2010, 18.1% attributed to smoking, with 19.1% absolute increase since 2010, < 1% attributed to occupational exposure to trichloroethylene, with 40.5% absolute increase since 2010^30^.

Another potential cause of the rising incidence is the higher utilization of imaging and discovery of incidental small renal masses, which account for nearly half of the new cases of renal cell carcinoma^31^. Detection of lower stage disease is associated with a better prognosis, and therefore the earlier detection of KC, as per the stage migration phenomenon, may be responsible for some of the improvements in patient outcomes^32^. Asymptomatic masses are more frequently diagnosed given the increased clinical use of modern imaging such as abdominal CT scans for evaluating urological and non-urological symptoms^11,33^. Indeed, a study in Canada showed an annual increase of CT imaging rates by 11.6% between 2000 and 2006, which decreased to 3.7% from 2013 to 2016^34^. This is comparable to our results where Canada had an EAPC of ASIR of 4.4 for females in the years 1998–2009 and 5.9 for males in the years 2001–2004, compared to a lesser value of -1.6 in the years 2006–2016 for females and -0.1 for the years 2008–2019 in males. Similarly, CT use continuously increased for assessing abdominal pain in the emergency departments of the United States from 1997 to 2016, concurring with an increased ASIR for males in the USA^35^. The increased frequency of radiological diagnosis would not be the sole reason behind the rise in ASIR in low-income countries since it has been demonstrated that these countries do not have equal access to imaging modalities compared to high-income countries^36^. An improvement in healthcare access is noted in the Middle East and North Africa (MENA) region in the past few decades with a rise in life expectancy at birth from 65 years in 1990 to 71 years in 2012, which could reflect on the increased diagnosis of KC^37^. However, significant disparities among and within countries still exist, especially with the unregulated intervention of the private sector to fill the gaps of the governments’ insufficient coverage, raising concern for equity^37^. Thus, a multifactorial model would be the ultimate explanation for the increased KC incidence in different countries.

Lower MIRs worldwide may be partly due to the stage migration with the detection of lower stage disease on cross-sectional imaging. These tumors are associated with a lower likelihood of cancer mortality, and 30% were indolent on post-operative pathology^38,39^. However, a recent article highlighted a paucity of imaging modalities in low to middle-income countries. Thus, over-diagnosis is not the only explanation for the universal drop in MIR^36^. We showed that mortality is also decreasing in most of the included countries. One plausible explanation for the mortality drop would be the approval of 10 novel drugs by the US Food and Drug Administration (FDA) for the treatment of metastatic RCC between 2005 and 2016, with the first approved targeted therapy being Sorafenib, a tyrosine kinase inhibitor (TKI)^40,41^. The inclusion of targeted therapies in the therapeutic arsenal of KC was proven by several studies to positively impact patients’ survival^42–44^. Our results mirror these findings as we noticed a drop in EAPCs of ASMRs from 2005 onwards, during the era of targeted therapies, in the high-income and upper-middle-income classes in the World Bank classification. On the other hand, EAPCs of ASMRs were still increasing after 2005 in middle-low to low-income countries, possibly due to a lack of access to comprehensive multidisciplinary team care, clinical trials, and advanced treatment modalities such as stereotactic radiotherapy medicine^45^. Additionally, now a subset of patients with metastatic RCC can be cured with immune-oncology (IO-IO) combinations but these recently entered into practice in 2018^46^. Therefore, future analysis will give information about its effects on survival and mortality. Poor access to treatment and imaging modalities plays a role in increased mortality rates in such regions since tumors are diagnosed at a later stage and are treated in a suboptimal manner^45^. Indeed, we highlighted that middle and low-income countries had higher increases in ASMRs and smaller drops in MIR than high-income countries. Similarly, a previous article had comparable conclusions to ours, having found a positive correlation between the availability of imaging examinations/effective systemic therapies in high-income countries and a favorable prognosis; the authors also concluded that MIRs were negatively correlated with the human development index and the current health expenditure per capita^47^. In this setting, Fay et al. emphasized the importance of clinical trial enrollment as a solution to health disparities by concluding to a similar overall survival among patients with metastatic RCC recruited in clinical trials across different geographic regions, despite different baseline characteristics^48^. We observed that the DALYs were decreasing in most of included Western European countries, especially in females. However, the DALYs remained stable in the European region. This is probably attributed to an increase in DALYs in Eastern Europe as opposed to a decrease in Western Europe^49^.

Finally, as we demonstrated an increase in incidence and mortality from KC in most included countries and regions, future focus is needed to implement strategies for the detection of early-stage KC. Extra efforts need to be put into decreasing the prevalence of smoking, obesity, and hypertension, which are among the strongest risk factors of KC, through campaigns and medical attention^50,51^. Although MIRs are decreasing, thus indicating an improvement in outcomes, equal access to treatment would ameliorate even more the situation worldwide, especially by using novel targeted and IO therapies^51^.

## Limitations

Limitations following the use of the GBD database were noted previously by our group and the GBD Study collaborators^15,16,52^. Regarding this study, the first limitation is the presence of alterations in data coding systems and country-specific practice during the study period, markedly a shift from the use of ICD 9 to ICD 10. However, the GBD authors map mortalities to causes of death lists, adjusting by such to the different coding systems. Secondly, variability in the reliability of death certification exists both within and across countries, with worldwide errors in death certification ranging from 39 to 61%^53–55^. Also, only 39% of deaths globally were registered in 2012. Considering that Europe, the Americas, and Australasia were ranked among the best continents with civil registration and vital statistics^56^, we demonstrated valid data of the EU15 + countries and 6 WHO regions assessed. To balance the under-registration, the GBD uses garbage-code distribution algorithms and corrections^17,18^, which relate to deaths resulting from poorly defined diagnoses or those that cannot be the single underlying cause of death. Thirdly, we could not extract the subcategorized data by individual KC histological subtypes from the GBD Study results tool, which should be considered when interpreting the results. Indeed, histopathological subtypes and stages of KC amend to different clinical significance and urgent management. Finally, since our study is an observational analysis of the trends in the burden of KC across 29 years in EU15 + countries and 6 WHO regions, causal inferences cannot be concluded. Moreover, as seen in observational analyses, some potential confounders were not accounted for by using sex-specific, age-standardized incidence and mortality rates.

## Conclusions

While the incidence and mortality from KC rose in most EU15 + countries and WHO regions from 1990 to 2019, the universal drop in MIR suggests an overall improvement in KC outcomes. This is likely multifactorial, including earlier detection of KC and improved treatments. These results are also of interest from a public health perspective as they highlight the importance of adjustable risk factor modification. A further study among lower-income countries would help identify the burden of disease across these regions and subsequently enable appropriate strategies to be developed to bridge disparities across countries and optimize patient care.

## Data Availability

The datasets analysed during the current study are available in the GBD repository, http://ghdx.healthdata.org/gbd-results-tool.

## Abbreviations

ASIR: Age-standardized incidence rate
ASMR: Age-standardized mortality rate
CT: Computed tomography
DALYs: Disability-adjusted life years
EAPC: Estimated annual percentage change
EU: European Union
GBD: Global burden of disease
ICD: International classification of diseases
IO: Immuno-oncology
KC: Kidney cancer
RCC: Renal cell carcinoma
UK: United Kingdom
USA: United States of America
WHO: World Health Organization
